# Management of Metachronous Bilateral Testis Cancer in a Patient with Pre-B Cell ALL

**DOI:** 10.1155/2015/646875

**Published:** 2015-02-03

**Authors:** Kelly T. Harris, Shakil A. Shaikh, Mark W. Ball, Mohamad E. Allaf, Phillip M. Pierorazio

**Affiliations:** The James Buchanan Brady Urological Institute and Department of Urology, Johns Hopkins University School of Medicine, Baltimore, MD 21287, USA

## Abstract

We present a patient with a metachronous, second testicular cancer after being diagnosed with pre-B cell ALL and receiving induction chemotherapy for a bone marrow transplant. We discuss the management of bilateral testis masses in a young patient with a hematologic malignancy as well as the role of immunosuppressive chemotherapy in developing a second cancer. This case illustrates the importance of recognizing bilateral testicular cancer early, as well as the importance of follow-up care in oncology patients including routine measurements of tumor markers. A multidisciplinary approach between medical oncology and urology, including close monitoring of the contralateral testis, remains paramount to patient care.

## 1. Introduction

Testicular cancer is the most common cancer of young men and its incidence continues to rise, with current estimates of 5.3 cases per 100,000 men [1, 2]. The development of bilateral testis cancer is exceedingly rare, estimated to be between 1 and 5% of patients with testis cancer [3]. Although bilateral testis cancer is rare, a previous history of testis cancer is the greatest risk factor, with the contralateral testicle having a 25-fold increased risk of malignancy [4].

Men with a history of hematologic malignancy are uniquely predisposed to recurrence of testicular cancer due to chemotherapeutic immunosuppression. In patients with acute lymphoblastic leukemia (ALL), Koike et al. found that relapse of testis cancer is common in both children and adults after an induction chemotherapy and bone marrow transplant [5]. However, discovery of an independent, second testicular primary with simultaneous leukemic involvement proves to be rare. It is much more likely to have hematogenous spread and invasion of the testis than to find a primary testicular tumor.

In this report, we present a patient with a metachronous, second testicular cancer after being diagnosed with pre-B cell ALL and receiving induction chemotherapy for a bone marrow transplant. We discuss the management of bilateral testis masses in a young patient with a hematologic malignancy as well as the role of immunosuppressive chemotherapy in developing a second cancer. This case illustrates the importance of recognizing bilateral testicular cancer early, as well as the importance of follow-up care in oncology patients including routine measurements of tumor markers.

## 2. Case Report

A nineteen-year-old Caucasian male originally presented to an outside hospital with right testicular pain that was sudden in onset and persistent in nature for three-month duration. Scrotal examination revealed a firm and hard right testicle. Scrotal ultrasound demonstrated a heterogeneous mass in the right testis, consistent with malignancy. Testicular tumor markers were elevated, with an alpha-fetoprotein (AFP) level of 9.3 ng/mL and a beta-hCG level of 3.1 mIU/mL. These radiologic and laboratory findings prompted surgical management with right radical orchiectomy.

Final pathology demonstrated a gray-white tumor with multiple cysts of varying size and a focal area of hemorrhage. The tumor was found to be a 3.0 × 2.0 × 2.0 cm nonseminomatous germ cell tumor (90% teratoma, 8% yolk sac tumor, and 2% embryonal carcinoma), which was confined to the testicle and without lymphovascular invasion (staging: pT1NxMxS0).

Postoperative management options were discussed, which included surveillance, primary chemotherapy, and retroperitoneal lymph node dissection (RPLND). He ultimately elected to undergo laparoscopic RPLND at our institution. Final pathology demonstrated eighteen lymph nodes and associated fibroadipose tissue negative for tumor. Follow-up CT scans showed small mesenteric adenopathy of the lower abdomen, but no evidence of bulky retroperitoneal lymphadenopathy or metastatic involvement. The patient recovered well, had normal antegrade ejaculation, and returned to his normal activities.

Three years later, he presented to the emergency room with blurred vision, fatigue, and easy bruising. He also complained of palpitations, cramping in his hands and legs, and a recent twelve-pound weight loss over the past month. A complete blood count showed a white blood cell count of 115,000/mcL, hematocrit of 17%, and platelets of 8,000. On physical exam, he was found to have bilateral intraretinal hemorrhages, bilateral papilledema, Roth's spots, splenomegaly, and bruises seen on fingernail beds and chest. Genitourinary exam showed mild pain in the left testicle with no swelling. He was ultimately diagnosed with a high-risk pre-B cell ALL with CNS II involvement.

Flow cytometry from the patient's bone marrow aspirate showed 89% abnormal cells. These cells were mostly blasts and found to be CD10 negative, CD19 positive, CD22 positive, and partial CD34 positive, consistent with an unusual precursor-B cell ALL. Kappa and lambda were negative and myeloid antigens were expressed. The patient's cytogenetic studies also showed AFF1/MLL fusion, consistent with a *t*(4; 11) MLL. The AFF1/MLL fusion is reported to be associated with an unfavorable prognosis of pre B-cell ALL.

**Figure 1:**
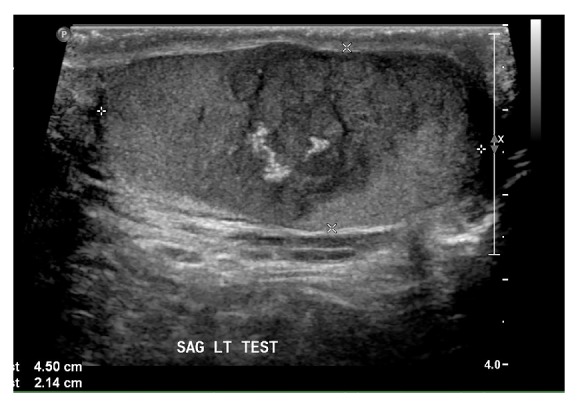
Sagittal sonogram of the left testis demonstrates testicular mass with calcifications.

The patient completed induction chemotherapy according to AALL1131 protocol, which consisted of one dose of intrathecal cytarabine, prednisone on days 1–28, daunorubicin on days 1, 8, 15, and 22, vincristine on days 1, 8, 15, and 22, pegaspargase on day 4, and intrathecal methotrexate on days 8 and 29 if blasts were continually seen in his CSF. After completion of this regimen, he returned for a haploidentical bone marrow transplant. At this visit, screening labs were drawn, which showed an incidental finding of an elevated beta hCG of 48.2 mIU/mL. Testicular ultrasound showed a 2- to 3-centimeter irregularly shaped hypoechoic mass in the left testicle, with associated microcalcification and increased vascularity (Figure 1). It was not known whether this represented metastasis of his hematologic malignancy or a primary testis malignancy.

Because the mass was in a central location, hypervascular, and with distinct borders, its appearance was most consistent with a recurrent nonseminomatous germ cell tumor rather than an infiltrative hematologic process. Preoperative consultation included discussion of partial or radical orchiectomy including implications for fertility and hormone replacement therapy. The patient elected radical orchiectomy with prosthesis implantation due to concerns for malignant progression. Surgical pathology indicated a 3 cm mixed germ cell tumor that was 95% embryonal carcinoma and 5% yolk sac tumor in origin with direct invasion into the epididymis and with lymphovascular involvement (pT2NxMxS0; Stage IB). Postoperatively, his alpha-fetoprotein and beta hCG returned to normal limits within four weeks.

The patient received his bone marrow transplant ten days after the orchiectomy. Continued management has included routine measurements of tumor markers and serial CT imaging for surveillance of both malignancies.

## 3. Discussion

Evidence based management of bilateral testicular cancer is scant, particularly guidelines for surveillance for metachronous tumors. Of the risk factors for developing testis cancer including cryptorchidism, family history, past medical history of testicular cancer, and intratubular germ cell neoplasia, a history of testis cancer confers the highest risk of developing future, contralateral cancer [2, 3, 6, 7]. Up to 5% of men will develop a second primary contralateral testis tumor and about 65% will be metachronous. Men with metachronous tumors are on average three years younger than men with synchronous tumors [2, 8]. Most metachronous cancers have the same histology as the initial primary tumor: if the initial primary is seminoma, 68.9% of the second primary will be seminomatous, and if the initial primary is NSGCT, the second tumor will be NSGCT 55.4% of the time [2]. The time to recurrence also varies by histology, with seminomatous histology occurring earlier more frequently than NSGCT [8–12]. Most men with metachronous tumors are diagnosed as clinical stage I (73.3%), in comparison to men with synchronous tumors, which are more likely to be stage II or III at diagnosis [2]. However, despite this, only 10.9% of men with metachronous tumors were enrolled in a surveillance protocol after orchiectomy [2]. Our patient presents a clinical scenario that highlights many of these findings but also poses unique questions regarding the best management options.

In this case, the patient was young at his initial diagnosis, developed a metachronous tumor within 3 years, and was found to have NSGCT in the second tumor, all of which are consistent with other cases in the literature. However, our patient is unique in that he was found to have the second testicular mass in the setting of a new diagnosis of leukemia. This complicates the situation because, in general, the appearance of a testis mass in the setting of a new hematologic malignancy more likely suggests malignant infiltration rather than a new primary testis tumor.

Determining whether the testis mass is a new primary or metastatic hematologic malignancy is of paramount importance because treatment differs significantly. For metastatic spread into the testicle, Quaranta et al. suggest that young men with acute myelogenous leukemia or ALL fare better with total body irradiation plus testicular boost prior to stem cell transplantation. They had a decreased incidence of Leydig cell dysfunction and testicular failure with this regimen [13]. A new primary testis tumor should be treated according to the NCCN guidelines [14] and does not include total body irradiation.

One of the more interesting aspects of this case is the emergence of the second testicular mass after induction chemotherapy. The particular chemotherapy protocol that our patient underwent is a randomized phase III trial of a combination chemotherapy regimen. Of the immunosuppressive and chemotherapeutic agents used, including prednisone, daunorubicin, vincristine, pegaspargase, and intrathecal cytarabine and methotrexate, none are used as first line treatments for NSGCT. Therefore, it is not surprising that this regimen did not have a protective effect on the development of a metachronous testicular tumor.

Furthermore, it is possible that the immunosuppressive effects of this induction chemotherapy made our patient more susceptible to developing a metachronous tumor. It is a well-known concept that immunosuppressed patients are more susceptible to malignancy and there are a number of small series and case reports that specifically demonstrate the development of genitourinary cancers in this population. Tremblay et al. noted that patients who underwent renal transplantation were increasingly susceptible to genitourinary and skin malignancies after treatment with cyclosporine [15]. Cancer incidence in the aforementioned patient population reached 12.2%, with a mortality rate of 54%.

Leibovitch et al. compared the development of testicular cancers in patients with AIDS versus immunosuppressed transplant patients and found that, regardless of the cause of immunosuppression, all patients should be treated according to standard therapy as indicated by tumor histology and stage of disease [16]. The standard treatment for a metachronous testis cancer is radical inguinal orchiectomy. Secondary treatments may include chemotherapy, retroperitoneal lymph node dissection, and radiation depending on the histology and stage of the second tumor. Outcomes are generally excellent for patients with metachronous disease, ranging from 90 to 95% durable overall survival [8].

In men with bilateral testis cancer, partial orchiectomy is an option that should be discussed, particularly in young men with concerns about lifelong androgen replacement and fertility. Lawrentschuk et al. described partial orchiectomy as an acceptable approach for patients with metachronous germ cell tumors due to the decreased morbidity of hypogonadism with maintenance of fertility [17]. However, patients treated with partial orchiectomy require closer follow-up and may need adjuvant treatment and androgen substitution. Given the recent diagnosis of a hematologic malignancy and the low likelihood of fertility given multiple rounds of chemotherapy, our patient strongly desired radical orchiectomy for the best chance of long-term cancer cure.

One additional consideration is the idea that metachronous bilateral testicular cancers can be detected at the time of the initial orchiectomy by performing a contralateral testis biopsy. Laguna et al. note that carcinoma in situ on biopsy can be treated via radiation therapy to prevent future recurrence and possibly avoid a second orchiectomy [18]. However, the long-term preservation of androgen function and fertility in these patients is not well described and the likelihood of developing a second testis cancer may be too low to justify routine contralateral biopsy.

## 4. Conclusions

Management of concurrent testicular cancer in the context of a hematologic malignancy continues to be a challenge. Fortunately, both unilateral and bilateral testicular cancers can be cured with appropriate management. Unfortunately, these patients must still face treatment for ALL, which carries a worse prognosis than testis cancer. A multidisciplinary approach between medical oncology and urology, including close monitoring of the contralateral testis, remains paramount to patient care.
